# Performance Evaluation of In Situ Prepolymerized TPU/SMC Composite-Modified Asphalt Mixtures with Aggregate Structure Optimization

**DOI:** 10.3390/polym18192397

**Published:** 2026-09-30

**Authors:** Jun Zhang, Naisheng Guo, Hongbin Zhu, Ping Chen, Zhichen Wang, Hang Su, Guangshuai Wu

**Affiliations:** 1 College of Transportation Engineering, Dalian Maritime University, Dalian 116026, China; 2 Intelligent Engineering Technology Research Center, Harbin Cambridge University, Harbin 150069, China; 3 Guangzhou City University of Technology, Guangzhou 510800, China

**Keywords:** in-situ prepolymerization, thermoplastic polyurethane, asphalt, asphalt mixture, pavement performance, styrene–maleic anhydride copolymer

## Abstract

Conventional thermoplastic polyurethane (TPU)-modified asphalt suffers from poor high-temperature performance, and the influence of aggregate skeleton structure on the performance of TPU-based modified asphalt mixtures remains insufficiently understood. TPU-modified asphalt and SMC-grafted TPU composite-modified asphalt were prepared via in situ prepolymerization, using base asphalt (BA) and SBS-modified asphalt as control binders. Four types of AC-16 asphalt mixtures were designed and systematically evaluated in terms of volumetric properties, low-temperature mechanical behavior, pavement performance, and aging resistance. The coarse aggregate void filling (CAVF) gradation was introduced to compare performance differences among mixtures with identical binders but distinct aggregate skeleton structures. The results demonstrated that TPU/SMC composite modification enhanced the high-temperature performance of asphalt mixtures while maintaining satisfactory low-temperature cracking resistance. The dynamic stability of the composite-modified mixture was over 60% higher than that of the base mixture, while its maximum flexural tensile strain at low temperature exceeded that of the conventional SBS-modified system. After aging, the freeze–thaw splitting strength retention rate of the composite-modified mixture reached 98.5%, and its aging resistance outperformed that of the SBS-modified mixture. CAVF gradation further improved the performance of TSMA mixtures, increasing fatigue life by 22.5% while enhancing rutting and moisture damage resistance. These findings demonstrate that CAVF-based aggregate structure optimization can further improve the pavement performance of TSMA mixtures and provide an experimental basis for material selection and gradation design of steel bridge decks and cold-region asphalt pavements.

## 1. Introduction

Asphalt pavement has long been recognized as the dominant paving type for highway transportation infrastructure owing to its advantages of satisfactory riding comfort, short construction period, and convenient maintenance [1,2]. For steel structure bridges serving as critical projects in road networks, asphalt mixtures are similarly utilized as the core load-bearing layer of deck pavement. Three mainstream asphalt paving systems have been established to date, namely Guss asphalt mixture, stone mastic asphalt mixture, and epoxy asphalt mixture [3,4]. Among these systems, Guss asphalt and stone mastic asphalt mixtures are commonly plagued by inadequate high-temperature stability and fatigue resistance [5]. Epoxy asphalt mixtures are restricted from large-scale popularization and application by highly import-dependent raw materials, high cost, and stringent construction requirements [3,6]. Accordingly, steel bridge deck paving materials that combine synergistic high- and low-temperature performance, low cost, and easy constructability are still being sought.

Polyurethane, a class of polymer elastomers with excellent mechanical strength, oxidation resistance, chemical stability, and wear resistance, has emerged as a highly promising asphalt modification material [7,8,9,10]. Notably, the price of polyurethane raw materials is approximately 15,000 CNY per ton, far lower than that of epoxy resin at around 55,000 CNY per ton, endowing it with remarkable economic advantages for large-scale application in the road engineering field [8]. Among various polyurethane materials, thermoplastic polyurethane (TPU) has attracted extensive attention due to its unique microphase separation structure. Rigidity and heat resistance are provided by its hard segments, while elasticity and low-temperature toughness are conferred by its soft segments [11,12,13]. The low-temperature toughness and fatigue resistance of TPU-modified asphalt with this distinctive structure are superior to those of conventional styrene–butadiene–styrene (SBS)-modified asphalt (SMA) [11,14,15]. In terms of the modification mechanism, isocyanate groups in polyurethane molecules can react with active functional groups such as hydroxyl and carboxyl groups in asphalt to form covalent bonds. Interfacial bonding is thus strengthened, and the colloidal stability of asphalt is improved [16,17,18,19].

However, the high-temperature rutting resistance of asphalt modified solely by TPU is generally inferior to that of SMA, which limits its application in steel bridge deck pavement [20]. This deficiency has been addressed in existing studies through physical blending with rock asphalt or nanoclay, but such physically blended systems are generally associated with poor compatibility and inadequate storage stability, and are often accompanied by compromised low-temperature performance [15,21]. Styrene–maleic anhydride copolymer (SMC) is a low-cost thermoplastic material bearing both rigid styrene segments and active maleic anhydride (MAH) groups in its molecular chains, by which the stiffness and rutting resistance of asphalt can be effectively enhanced [22]. On one hand, the interfacial compatibility between TPU and asphalt is improved by MAH groups via polarity matching. On the other hand, MAH groups can react with hydroxyl groups or residual isocyanate groups at the ends of TPU molecular chains to form ester or imide bonds. A branched crosslinked structure is thus generated, which coexists with the inherent hydrogen-bonded physical network of TPU [23]. Such a grafted network structure provides an effective route to overcome the inherent trade-off between high-temperature rigidity and low-temperature toughness of TPU-modified asphalt. Instead of increasing the hard-segment content of TPU, SMC grafting introduces chemically bonded rigid segments as reinforcing nodes within the TPU network, thereby enhancing high-temperature rheological performance and structural stability while maintaining the low-temperature toughness derived from TPU soft segments [24]. Furthermore, the polymerization and chain extension processes of TPU are completed directly in the asphalt medium through the in situ prepolymerization process. More active end groups can be retained, and more sufficient chemical crosslinking can be promoted compared with the conventional two-step method of blending prefabricated TPU particles [25].

Previous studies have demonstrated the potential of SMC-g-TPU-modified asphalt binders in improving binder rheological properties and aging resistance. However, how to effectively translate this polymer modification strategy from the binder level to asphalt mixture applications and further improve mixture-level pavement performance remains insufficiently explored [12,13,25]. In addition, traditional dense-graded mixture designs are characterized by a suspended aggregate structure, under which the performance potential of modified asphalt cannot be fully exploited under the high-stress service conditions of steel bridge deck pavement [26,27]. A skeleton-dense structure can be formed by the coarse aggregate void filling (CAVF) method, which exhibits superior load distribution performance [28,29,30]. However, the influence of CAVF-based aggregate skeleton optimization on the pavement performance of SMC-grafted TPU-modified asphalt mixtures remains insufficiently investigated.

To address the aforementioned research gaps, TPU-modified asphalt (ITMA) and TSMA prepared via the in situ prepolymerization process in previous work were adopted in this study, with base asphalt (BA) and SMA employed as control binders. Four types of AC-16 suspended dense asphalt mixtures, namely AC-BA, AC-SMA, AC-TMA and AC-TSMA, were prepared. Their volumetric indices, high-temperature stability, low-temperature crack resistance, moisture damage resistance, fatigue resistance, and aging resistance were evaluated through laboratory tests to clarify the regulation mechanism of the in situ prepolymerization and grafting processes on asphalt mixture performance. Furthermore, a skeleton-dense gradation was constructed based on the CAVF method to prepare CAVF-TSMA mixtures. Performance comparisons between AC-TSMA and CAVF-TSMA mixtures were conducted to evaluate the effect of aggregate skeleton optimization on the performance enhancement of TSMA mixtures. The findings of this study provide experimental data and theoretical reference for material selection and mix proportion optimization of steel bridge deck pavement and asphalt pavement in severe cold regions.

## 2. Materials and Methods

### 2.1. Materials

#### 2.1.1. Asphalt

Asphalt samples employed in this study included BA and SMA manufactured by Heilongjiang Haoyang Asphalt Co., Ltd., Suihua, China, as well as self-prepared ITMA and TSMA via the in situ prepolymerization method. The technical properties of the asphalt binders are summarized in Table 1. Both ITMA and TSMA were prepared with BA as the base binder. Prior to preparation, synthetic raw materials including polytetramethylene ether glycol (PTMEG), diphenylmethane diisocyanate (MDI), 1,4-butanediol (BDO), and SMC were separately subjected to dehydration drying and crushing pretreatment.

Both modified asphalt binders were synthesized via a stepwise in situ prepolymerization process, with identical protocols adopted for the initial reaction stage. Dehydrated PTMEG and MDI were first added to BA and sheared at a rotational speed of 3000 r/min at 150 °C for 0.5 h to complete the prepolymerization reaction. BDO chain extender was subsequently introduced, and shearing was continued at 150 °C and 3000 r/min for an additional 0.5 h, after which a polyurethane phase was preliminarily formed in the system. For ITMA, shearing was maintained for another 0.5 h under the same temperature and rotational speed conditions to achieve uniform dispersion of the TPU phase in the asphalt matrix. After the shearing process, samples were placed in a 140 °C environment for 2 h of curing to refine the molecular crosslinking structure and enhance both system stability and high-temperature performance. Hard-segment content (*C_h_*) and isocyanate index (*r*) are two key control parameters in TPU synthesis. Previous studies [31] demonstrated that at *C_h_* = 40%, *r* = 1.05, and a TPU dosage of 5%, the resulting TPU-modified asphalt achieved an optimal balance between high-temperature stability and low-temperature ductility, along with optimal rheological properties. Accordingly, this parameter combination was adopted in the present study for TPU synthesis to ensure comprehensive pavement performance of the modified asphalt.

TSMA was fabricated by incorporating SMC into the system with a preliminarily formed polyurethane phase for graft modification. A 60:40 mass ratio of TPU to SMC was selected because it has been documented to achieve a desirable compromise among tensile strength, elongation at break, and thermal properties [32,33]. To guarantee adequate SMC melting in the asphalt matrix and its participation in building the molecular network, the system was heated to 180 °C and maintained under high-speed shear mixing for 0.5 h. Previous rheological results showed that SMC grafting increased the rutting factor |G*|/sin*δ* of in situ TPU-modified asphalt by an average of 15.9% within 46–55 °C, indicating a clear enhancement in high-temperature deformation resistance. Meanwhile, the low-temperature cracking resistance remained comparable to that of the in situ TPU-modified system [25].

During preparation, the system was heated to 180 °C before SMC was added, and sheared at 3000 r/min for 0.5 h to ensure sufficient melting of SMC and completion of the graft reaction. Sufficient activation energy for the graft crosslinking reaction was supplied by the high-temperature environment, and system curing was accomplished simultaneously during the reaction process. Accordingly, no additional curing procedure was required after shearing preparation. Details of the preparation procedure have been documented in the previously published literature [25].

**Figure 1:**
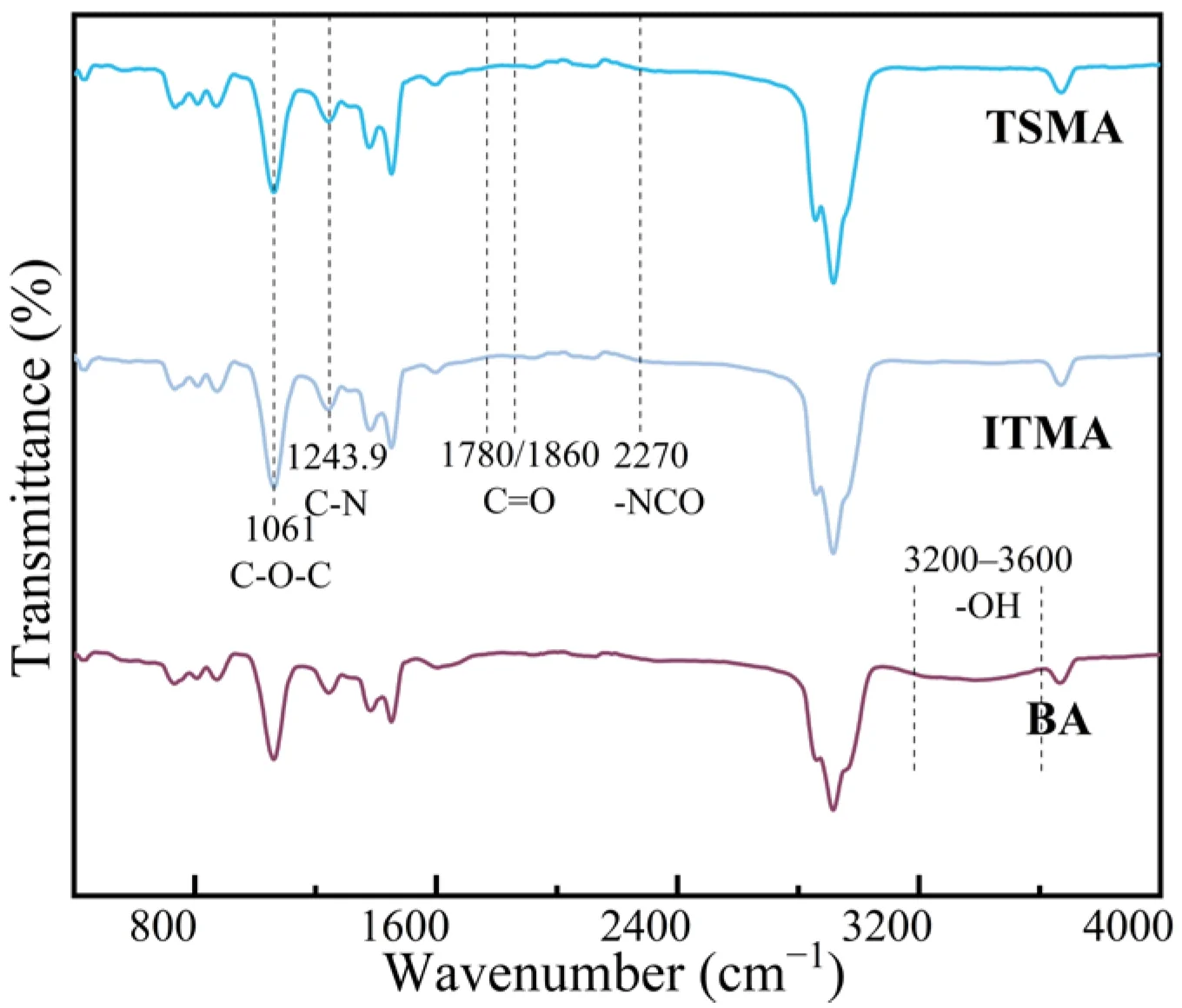
FTIR spectra of base asphalt and polymer-modified asphalt binders.

Fourier transform infrared spectroscopy (FTIR) analysis was further conducted to investigate the interactions among TPU, SMC, and asphalt components. As shown in Figure 1, compared with BA, the broad hydroxyl-associated band in the range of 3200–3600 cm^−1^ was weakened after TPU and TPU/SMC modification, which may be attributed to enhanced hydrogen-bonding interactions involving urethane groups (–NH– and C=O) and polar components in asphalt. Meanwhile, the integrated area of the C–N/N–H-related vibration band at approximately 1243.9 cm^−1^ increased by 27.0% and 14.6% for TPU- and TPU/SMC-modified asphalt, respectively. The C–O–C stretching band near 1060.9 cm^−1^ also exhibited an increase of more than 14%, corresponding to the contribution of the polyether soft segments in TPU. In addition, the increased intensity around 1604 cm^−1^ may be associated with the additional aromatic structures introduced by the MDI units in TPU. Furthermore, no obvious residual peaks corresponding to –NCO stretching vibration or anhydride carbonyl groups were observed in the modified asphalt binders, suggesting that most reactive groups were consumed during the in situ polymerization and grafting process, with limited residual unreacted species remaining in the system.

#### 2.1.2. Aggregate

Limestone crushed stone and manufactured sand supplied by Heilongjiang Longfeng Testing Center were adopted as aggregates in this study. The mineral aggregates were classified into six size fractions, namely 13.2~19 mm, 9.5~13.2 mm, 4.75~9.5 mm, and 3~6 mm crushed stone fractions, together with manufactured sand and mineral filler. Crushed stones used as coarse aggregates are required to possess a hard, dense texture, excellent abrasion resistance, no weathering or impurities, and dry, clean surfaces. Satisfactory adhesion between coarse aggregates and asphalt binder is also required. The technical properties of coarse aggregates are summarized in Table 2.

Manufactured sand derived from limestone parent rock was adopted as fine aggregate, with a particle size range of 0~3 mm. The fine aggregate was required to be clean and dry, with angular particles and rough surface texture, and free from organic soil and clayey silt. The technical properties of fine aggregates are presented in Table 3.

Ground limestone mineral filler was adopted as the mineral filler in this study. The technical properties of the mineral filler are presented in Table 4.

### 2.2. Mix Design of Asphalt Mixtures

#### 2.2.1. Design of AC-16 Mineral Aggregate Gradation

**Figure 2:**
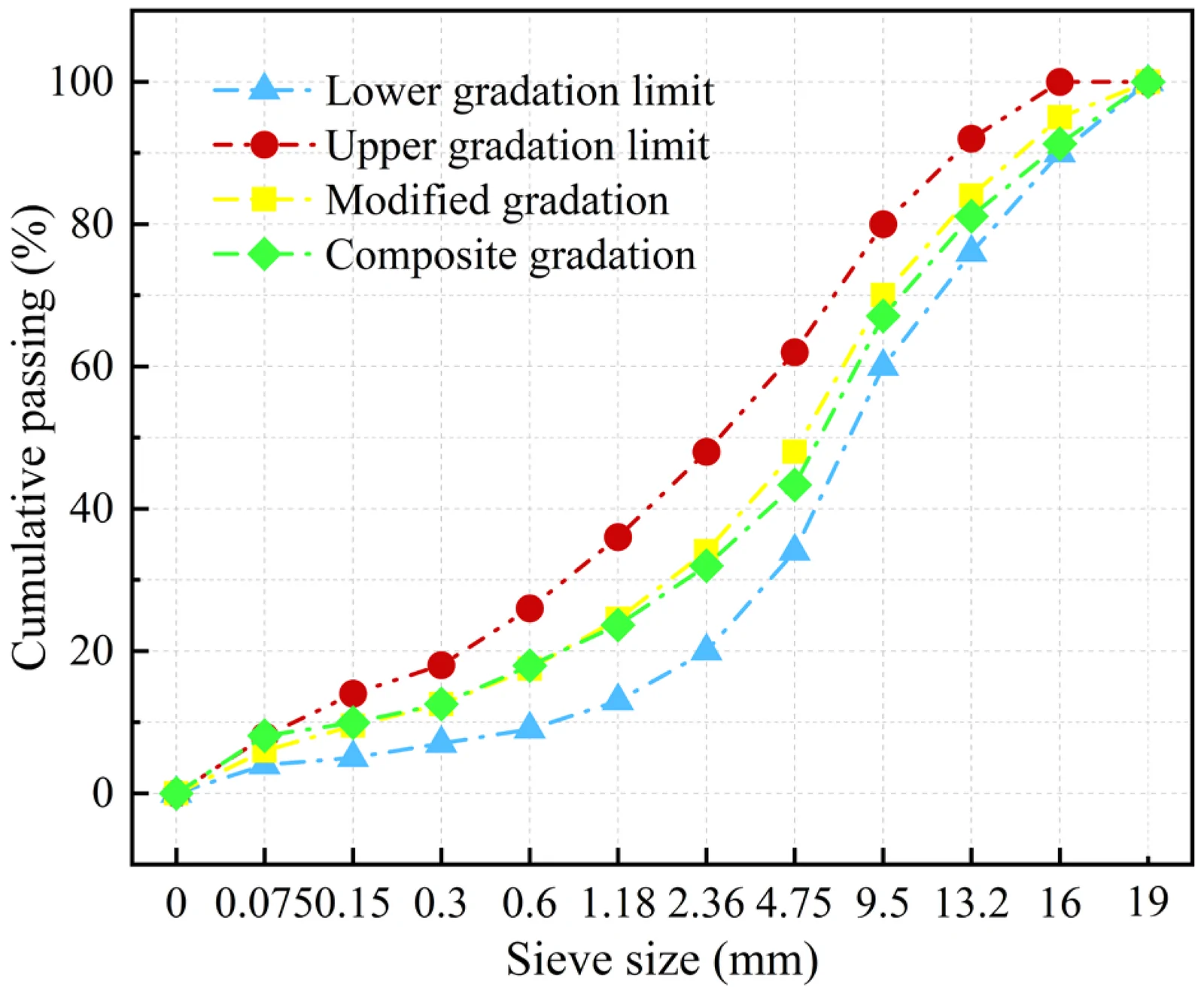
Gradation of the AC-16 asphalt mixture.

Gradation ranges for asphalt mixtures conforming to engineering design criteria are determined according to climatic conditions, traffic conditions, and highway grades. The AC-16 gradation was developed in compliance with the Technical Specification for Construction of Highway Asphalt Pavements (JTG F40-2004) to compare the volumetric parameters, mechanical properties, pavement performance, and aging resistance of four asphalt mixtures, namely AC-BA, AC-SMA, AC-TMA, and AC-TSMA. Table 5 presents the passing percentages of each aggregate fraction across all sieve sizes and the corresponding mixing proportions of each fraction. Figure 2 illustrates the gradation of the AC-16 asphalt mixture.

After gradation design was completed, specimens were fabricated via standard double-sided compaction with 75 blows per side in accordance with JTG F40-2004. Standard specimens were required to meet the height requirement of 63.5 ± 1 mm. Upon completion of all compaction processes, specimens were placed horizontally for 24 h of cooling and reserved for subsequent volumetric index tests. The optimum asphalt content (OAC) was subsequently determined based on the volumetric characteristics of the compacted specimens. For each asphalt content and mixture type, four parallel Marshall specimens were prepared, and the reported volumetric and Marshall indices represent the mean values of the four specimens.

(1) Determination of OAC for AC-BA

**Figure 3:**
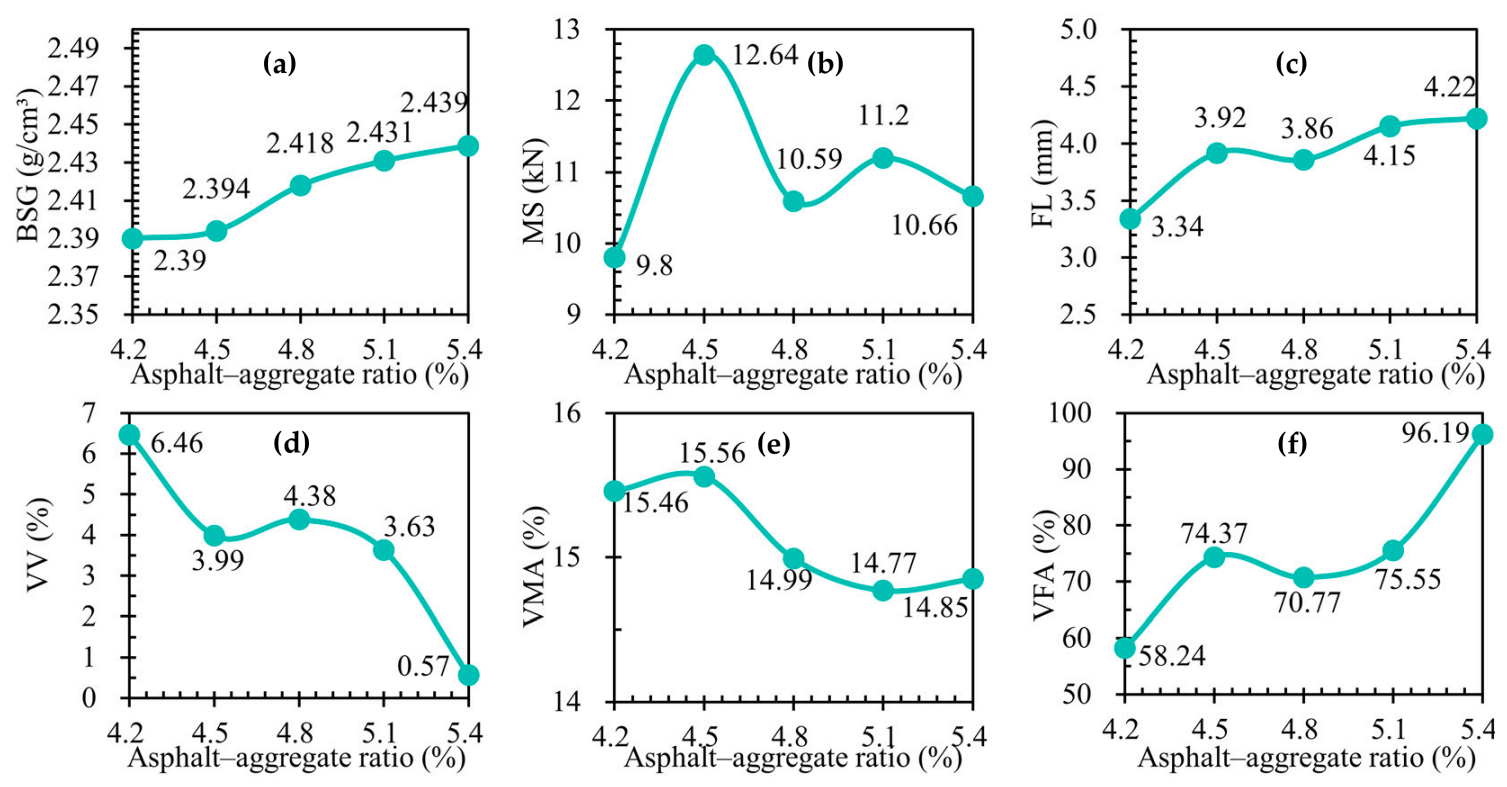
Results of the AC-BA Marshall test ((**a**), BSG; (**b**), MS; (**c**), FL; (**d**), VV; (**e**), VMA; (**f**), VFA).

**Figure 4:**
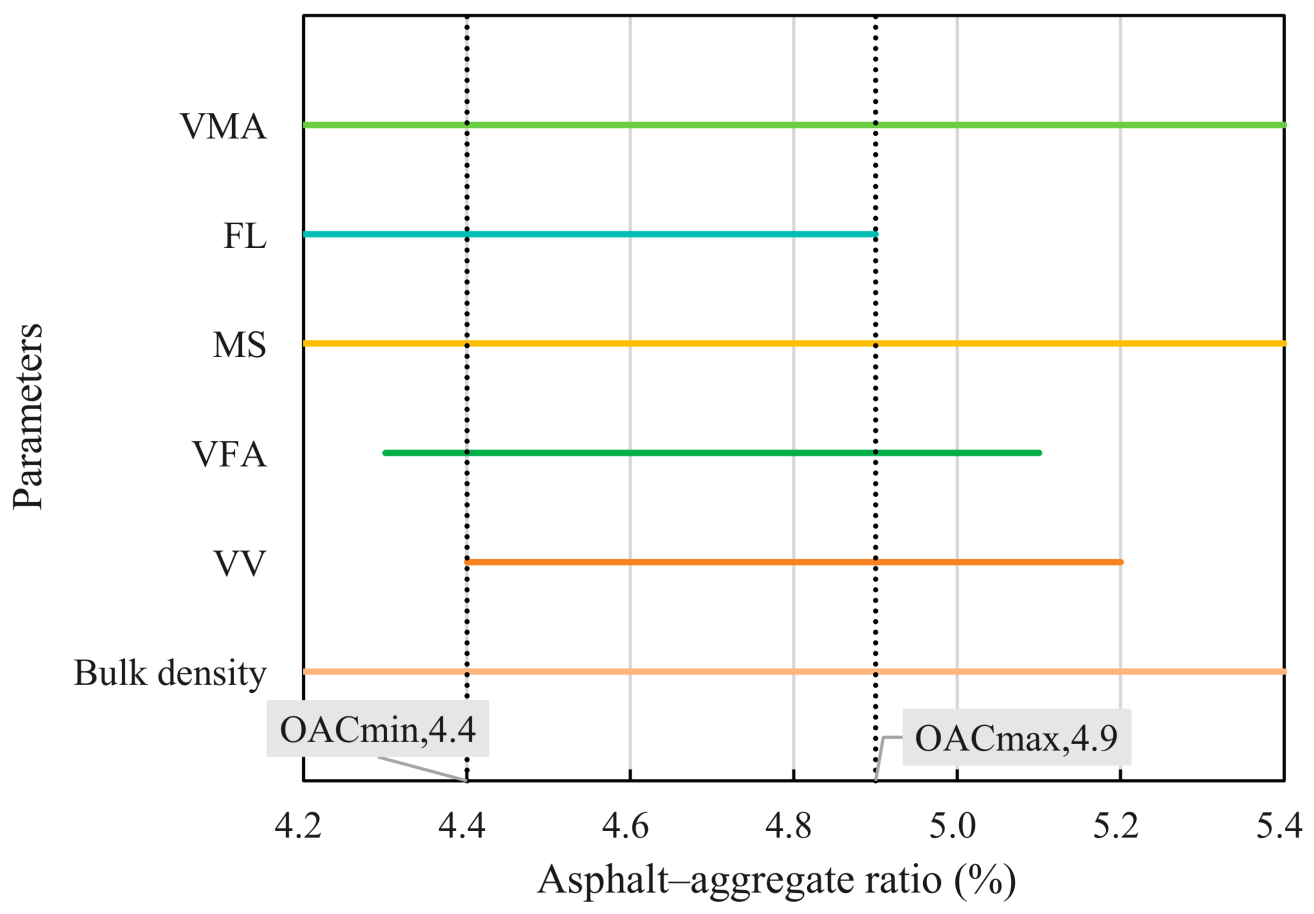
Determination of OAC_min_ and OAC_max_ for AC-BA.

Variation curves of BSG, air voids (VV), voids in mineral aggregate (VMA), voids filled with asphalt (VFA), Marshall stability (MS), and flow value (FL) for AC-BA versus asphalt–aggregate ratio were plotted, respectively, as illustrated in Figure 3. The minimum optimum asphalt–aggregate content (OAC_min_) and maximum optimum asphalt–aggregate content (OAC_max_) were determined based on the correlation between asphalt content and the physical and mechanical indices derived from the Marshall test, as presented in Figure 4.

An extreme value of index a1 for AC-BA was identified in accordance with the design specification, and the corresponding OAC1 was determined as 4.51%. The OAC_min_ of 4.40% and OAC_max_ of 4.90% were derived from the asphalt–aggregate ratio ranges specified in technical standards for each performance index. The OAC2 was calculated as OAC2 = (OAC_min_ + OAC_max_)/2 = 4.65%. The OAC was derived as OAC = (OAC1 + OAC2)/2 = 4.58%. After being rounded to two decimal places, the final OAC for AC-BA was set at 4.60%.

(2) Determination of OAC for AC-SMA

**Figure 5:**
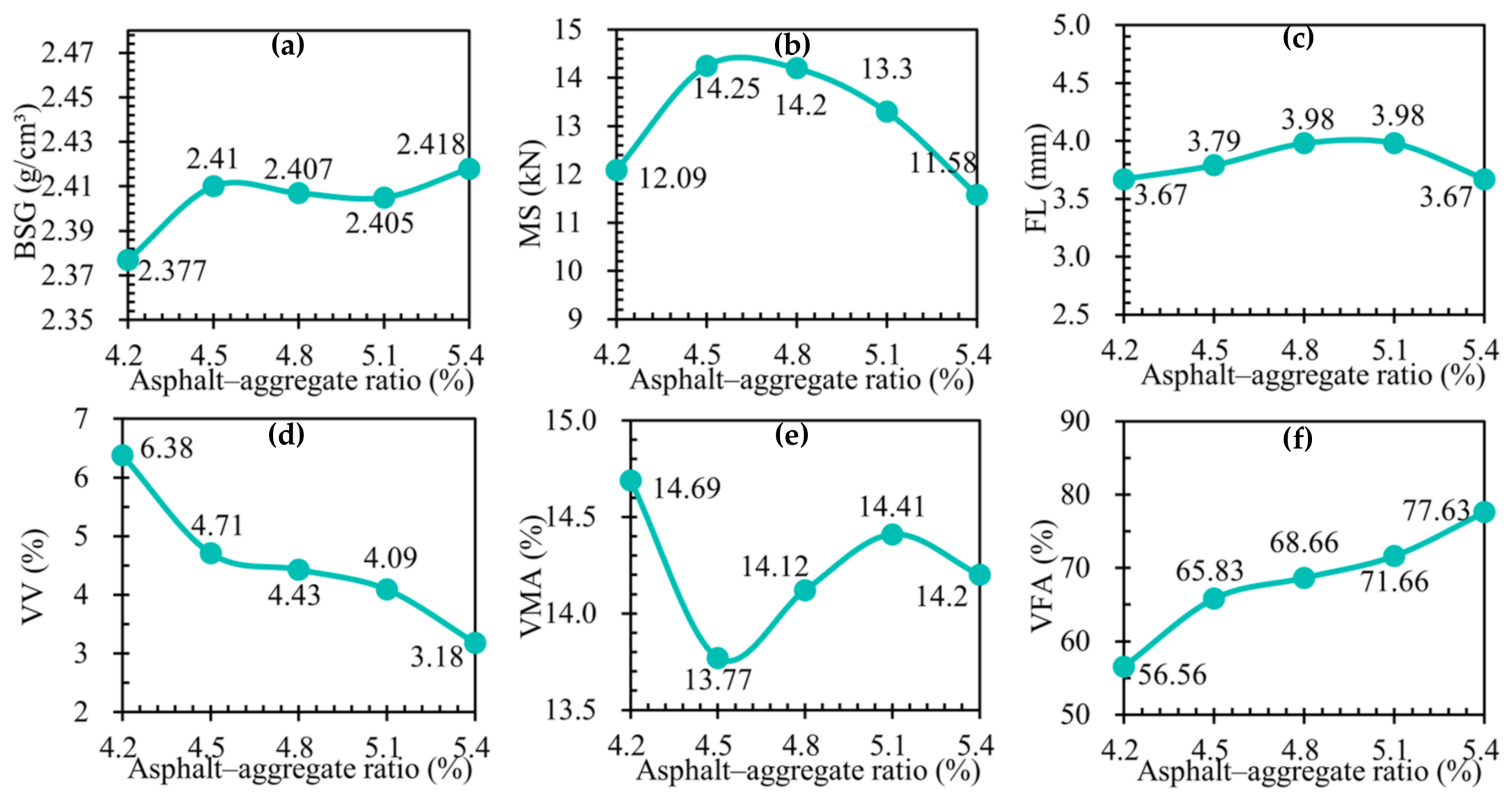
Results of the AC-SMA Marshall test ((**a**), BSG; (**b**), MS; (**c**), FL; (**d**), VV; (**e**), VMA; (**f**), VFA).

**Figure 6:**
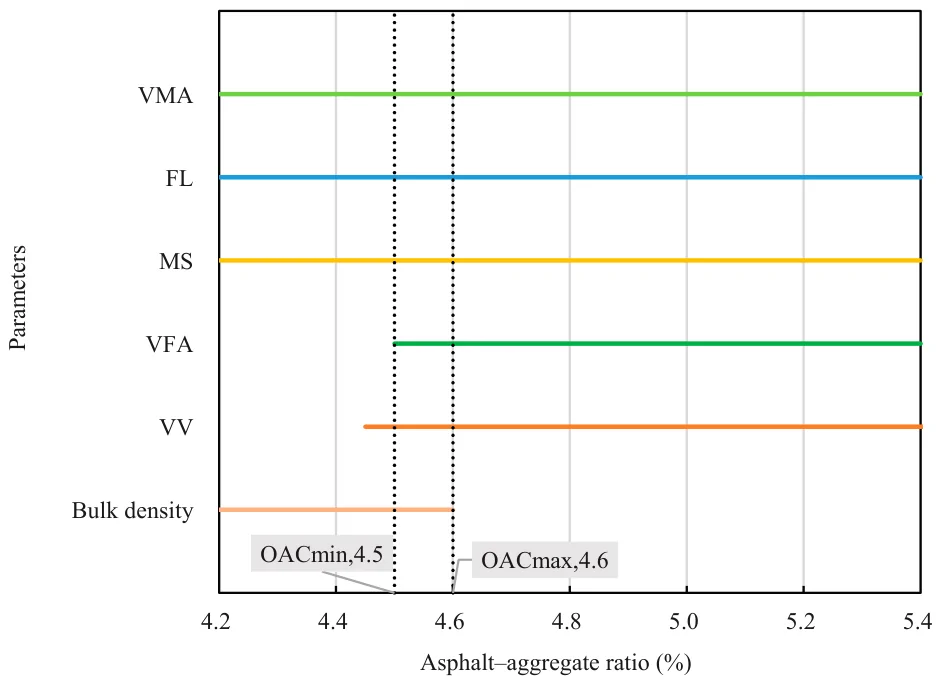
Determination of OAC_min_ and OAC_max_ for AC-SMA.

Variation curves of BSG, VV, VMA, VFA, MS, and FL for AC-SMA versus asphalt–aggregate ratio were plotted, respectively, as illustrated in Figure 5. The OAC_min_ and OAC_max_ were determined based on the correlation between asphalt content and the physical and mechanical indices derived from the Marshall test, as presented in Figure 6.

Four control index values for AC-SMA were derived from experimental results, with a1 = 4.58%, a2 = 4.60%, a3 = 5.13%, and a4 = 4.90%. Accordingly, OAC1 was calculated as OAC1 = (a1 + a2 + a3 + a4)/4 = 4.80%. The OAC_min_ of 4.50% and OAC_max_ of 4.58% were determined from the asphalt–aggregate ratio ranges specified in technical standards for each performance index. OAC2 was then computed as OAC2 = (OAC_min_ + OAC_max_)/2 = 4.54%. The OAC was derived as OAC = (OAC1 + OAC2)/2 = 4.67%. After rounding to two decimal places, the final OAC for AC-SMA was set at 4.70%.

(3) Determination of OAC for AC-TMA

**Figure 7:**
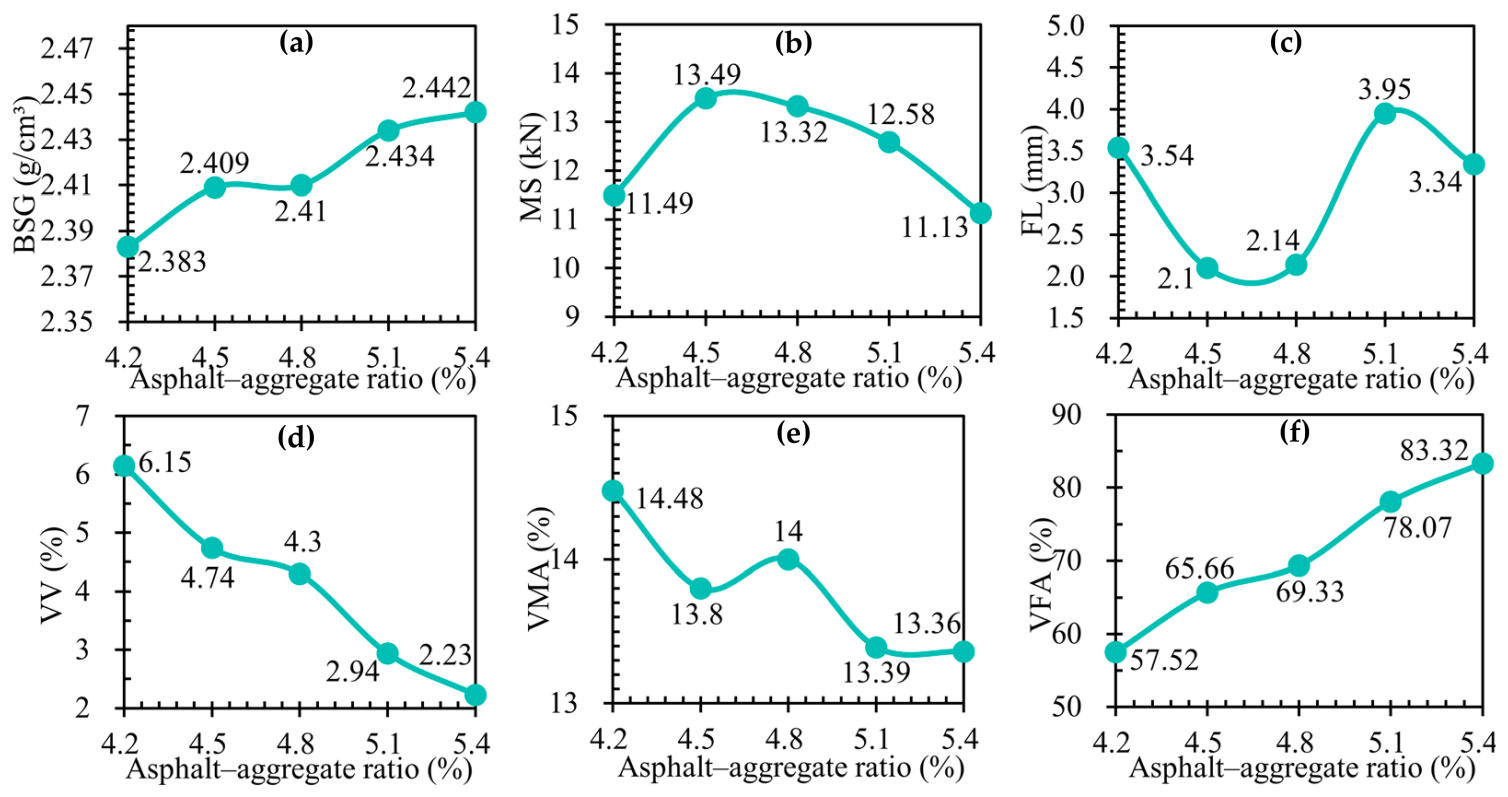
Results of the AC-TMA Marshall test ((**a**), BSG; (**b**), MS; (**c**), FL; (**d**), VV; (**e**), VMA; (**f**), VFA).

**Figure 8:**
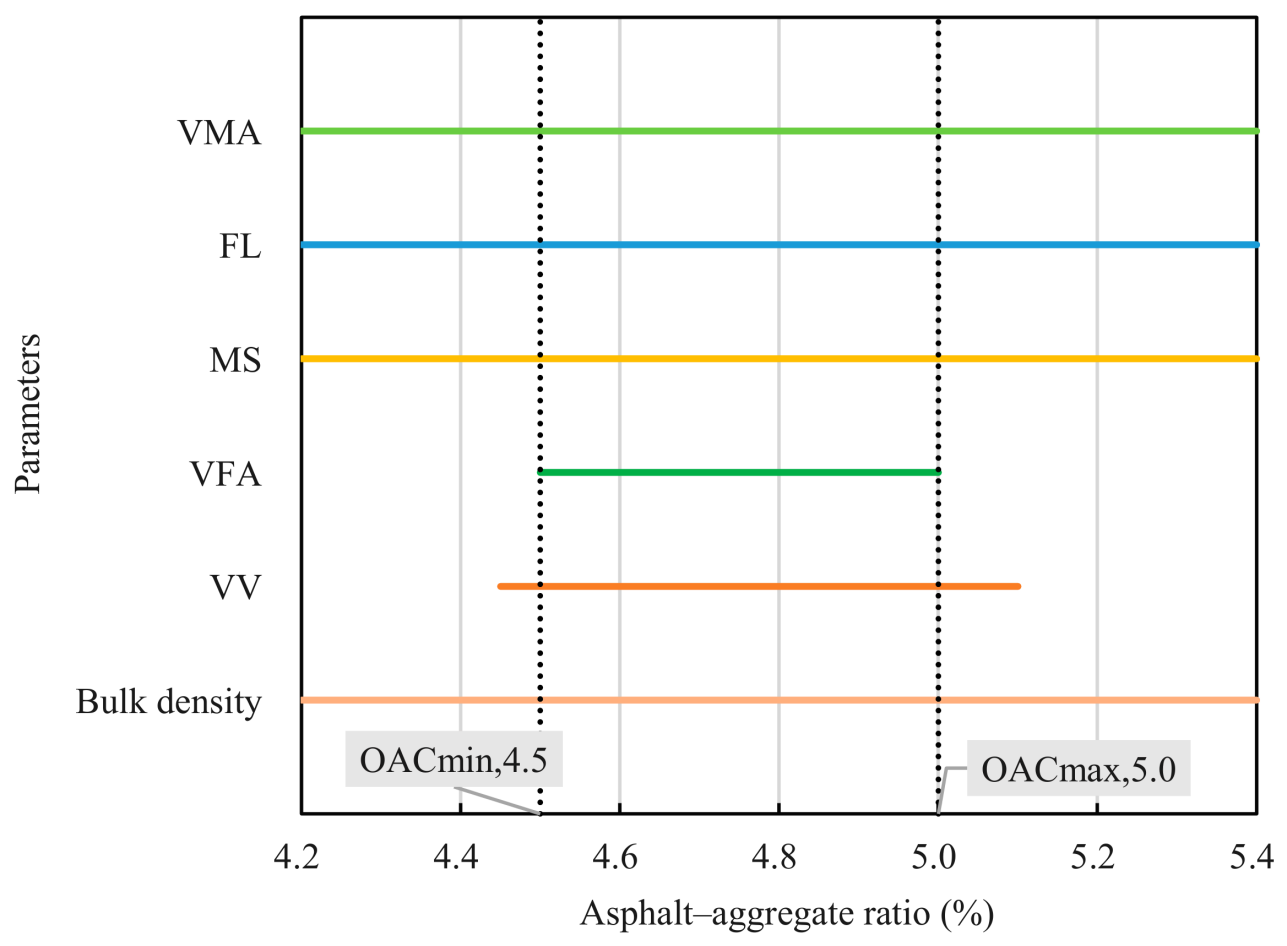
Determination of OAC_min_ and OAC_max_ for AC-TMA.

Variation curves of BSG, VV, VMA, VFA, MS, and FL for AC-TMA versus asphalt–aggregate ratio were plotted, respectively, as illustrated in Figure 7. The OAC_min_ and OAC_max_ were determined based on the correlation between asphalt content and the physical and mechanical indices derived from the Marshall test, as presented in Figure 8.

An extreme value of index a1 for AC-TMA was identified, and the corresponding OAC1 was determined as 4.87%. The OAC_min_ of 4.50% and OAC_max_ of 5.00% were derived from the asphalt–aggregate ratio ranges specified in technical standards for each performance index. OAC2 was calculated as OAC2 = (OAC_min_ + OAC_max_)/2 = 4.75%. The OAC was derived as OAC = (OAC1 + OAC2)/2 = 4.81%. After being rounded to two decimal places, the final OAC for AC-TMA was set at 4.80%.

(4) Determination of OAC for AC-TSMA

**Figure 9:**
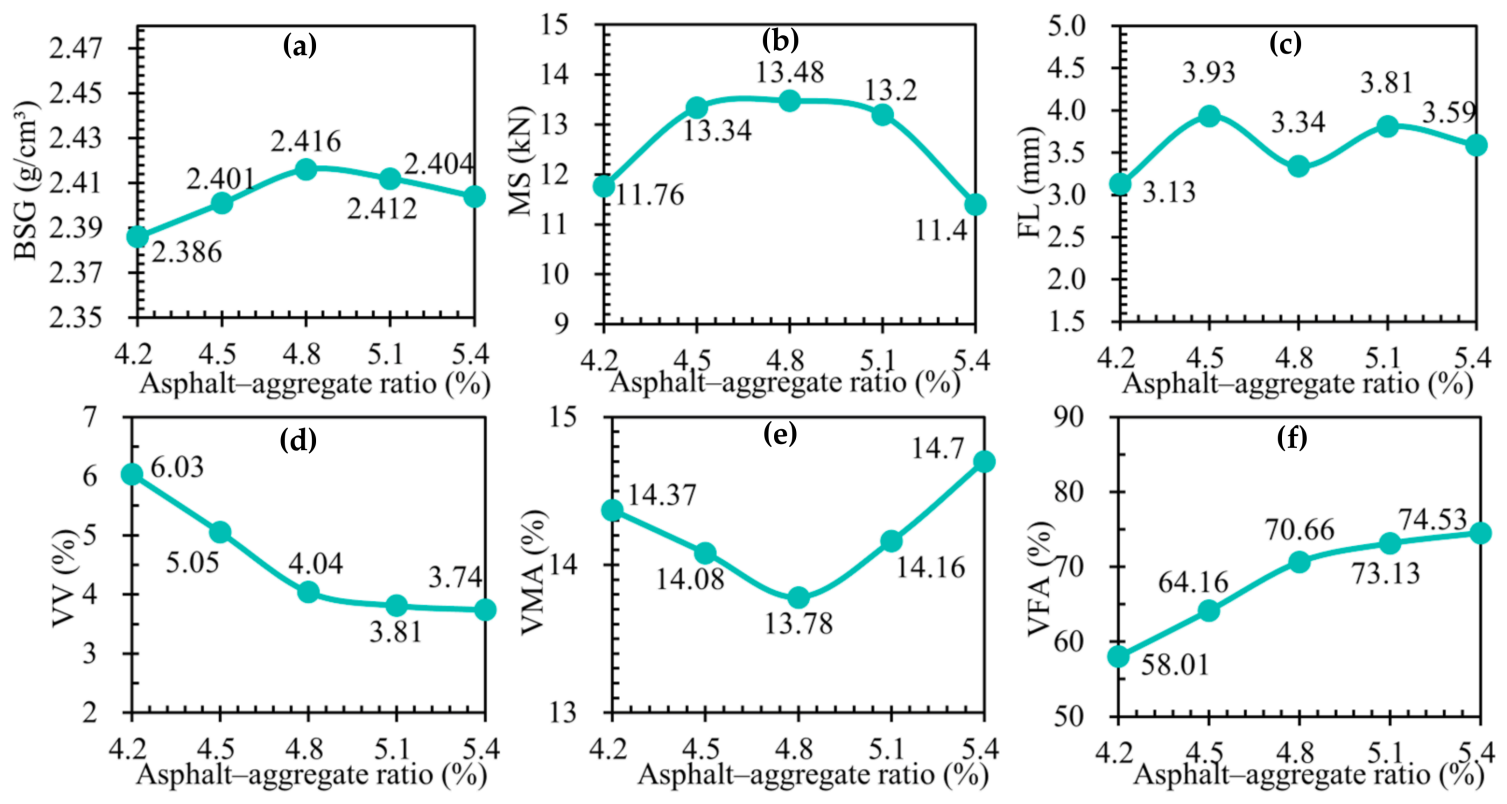
Results of the AC-TSMA Marshall test ((**a**), BSG; (**b**), MS; (**c**), FL; (**d**), VV; (**e**), VMA; (**f**), VFA).

**Figure 10:**
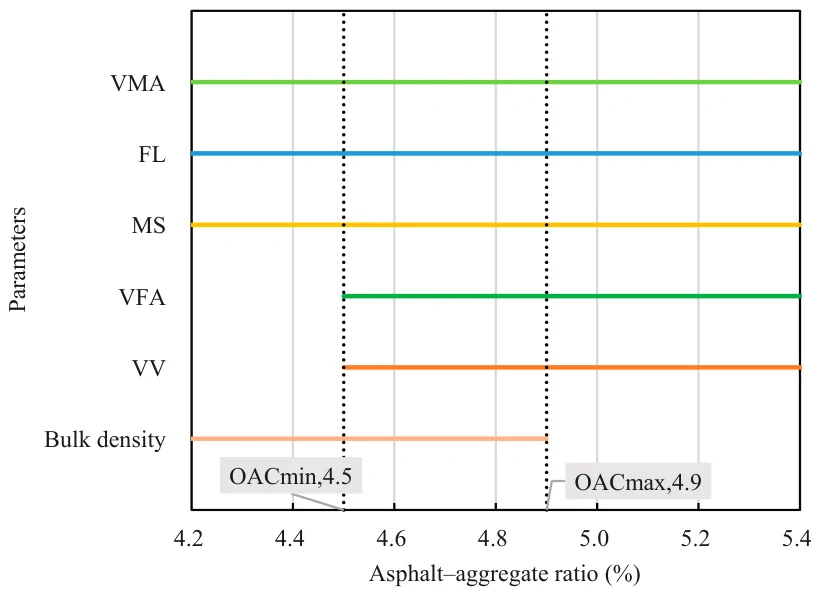
Determination of OAC_min_ and OAC_max_ for AC-TSMA.

Variation curves of BSG, VV, VMA, VFA, MS, and FL for AC-TSMA versus asphalt–aggregate ratio were plotted, respectively, as illustrated in Figure 9. The OAC_min_ and OAC_max_ were determined based on the correlation between asphalt content and the physical and mechanical indices derived from the Marshall test, as presented in Figure 10.

Four control index values for AC-TSMA were derived from experimental results, with a1 = 4.85%, a2 = 4.80%, a3 = 4.81%, and a4 = 4.80%. Accordingly, OAC1 was calculated as OAC1 = (a1 + a2 + a3 + a4)/4 = 4.82%. The OAC_min_ of 4.51% and OAC_max_ of 4.85% were determined from the asphalt–aggregate ratio ranges specified in technical standards for each performance index. OAC2 was then computed as OAC2 = (OAC_min_ + OAC_max_)/2 = 4.68%. The OAC was derived as OAC = (OAC1 + OAC2)/2 = 4.75%. After rounding to two decimal places, the final OAC for AC-TSMA was set at 4.80%.

#### 2.2.2. Design and Preparation of CAVF-TSMA Mixture

To construct a stable coarse aggregate skeleton and improve the compatibility between polymer-modified asphalt binder and aggregate structure, the CAVF method was employed to design the TSMA mixture. The CAVF design concept aims to optimize the packing structure of coarse aggregates and ensure sufficient filling of the skeleton voids by asphalt mastic. The aggregate skeleton was initially designed using the V-S multi-point supported skeleton model. The theoretical proportions of coarse aggregate fractions were determined according to the particle interference relationship between adjacent aggregate sizes. The calculation was performed using Equation (1):

(1) $$V_{i}=\frac{V_{i-1}}{{\left(1+\frac{d}{D}\right)}^{3}}$$

where *V_i_* represents the residual solid volume after introducing smaller aggregate particles, while *D* and *d* represent the characteristic particle sizes of the larger and smaller aggregate fractions, respectively.

**Figure 11:**
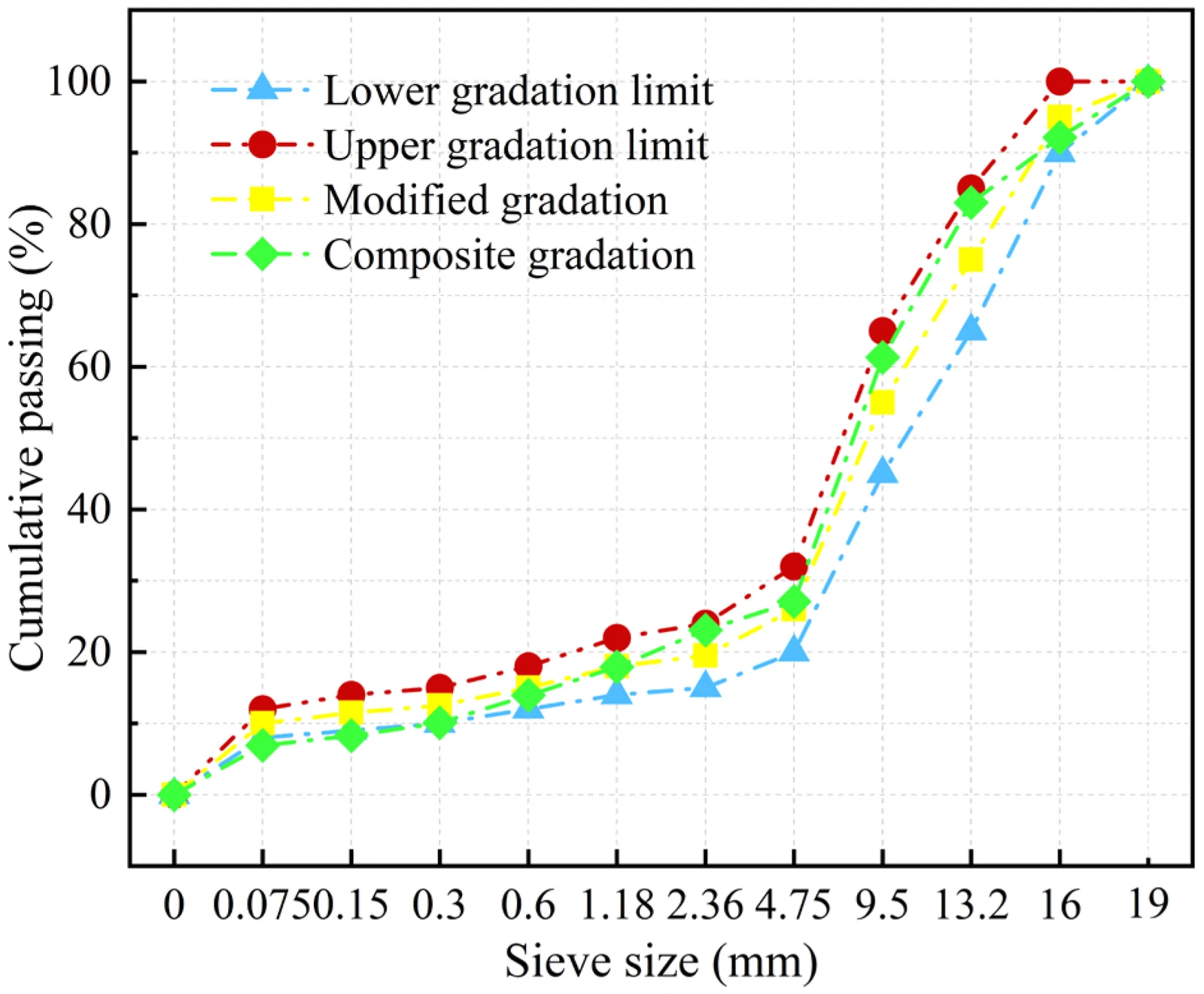
Gradation of the CAVF-TSMA mixture.

Subsequently, the modified CAVF method was introduced by incorporating an interference coefficient *α* to consider the influence of fine aggregates and asphalt mastic on the coarse aggregate skeleton. In this study, *α* was selected as 0.9. The modified skeleton structure increased the proportion of coarse aggregates and reduced the required amount of fine aggregates, facilitating the formation of a stable coarse aggregate framework. Considering the requirements of gap-graded SMA mixtures, the 3–6 mm aggregate fraction was excluded and the mineral filler content was fixed at 4%. Based on the theoretical coarse aggregate distribution obtained from the V-S model, the modified coarse-to-fine aggregate proportion, and the actual stockpile gradations, the aggregate fractions were further adjusted to obtain the final CAVF gradation. The final stockpile proportions were 18%, 24%, 33%, 0%, 21%, and 4% for the 13.2–19 mm, 9.5–13.2 mm, 4.75–9.5 mm, 3–6 mm, manufactured sand, and mineral filler fractions, respectively. The corresponding combined gradation curve is presented in Figure 11.

The optimum asphalt content of the CAVF-TSMA mixture was determined through Marshall volumetric design. Five asphalt contents ranging from 4.2% to 5.4% were evaluated, with four parallel Marshall specimens prepared at each asphalt content. The bulk density, Marshall stability, air voids, voids in mineral aggregate, and voids filled with asphalt were determined from the mean values of the corresponding specimens. The characteristic asphalt contents corresponding to the maximum bulk density, maximum Marshall stability, designed air void content, and target asphalt saturation were determined as 5.06%, 4.50%, 5.40%, and 5.00%, respectively. Accordingly, the preliminary optimum asphalt content calculated by the volumetric method was 4.99%. Considering the volumetric characteristics and the asphalt mastic filling requirement of the CAVF skeleton, the final OAC of the CAVF-TSMA mixture was selected as 5.00%.

### 2.3. Test Methods

#### 2.3.1. Pavement Performance Tests

Wheel tracking, low-temperature bending, and freeze–thaw splitting tests were conducted on the five asphalt mixtures following procedures T0719, T0715, and T0729 specified in JTG 3410-2025, respectively. Three parallel specimens were tested for each mixture in the wheel tracking test, and four valid beam specimens were tested in the low-temperature bending test. For the freeze–thaw splitting test, eight specimens per mixture were equally divided into unconditioned and freeze–thaw-conditioned groups. Fatigue life was evaluated using a semicircular bending (SCB) test with three specimens per mixture. The SCB specimens were 50 mm thick, with a 10 mm deep and 1.5 mm wide notch, and the support span was 120 mm. Tests were performed at 15 °C under stress-controlled cyclic loading at 10 Hz and a stress ratio of 0.3. Complete fracture was taken as the failure criterion. The reported results are the mean values of the corresponding replicate specimens.

#### 2.3.2. Accelerated Aging Test of Asphalt Mixtures

Asphalt mixtures are susceptible to thermal aging during mixing, transportation, and paving. After the pavement is constructed and put into service, the asphalt binder continues to age under long-term actions including ultraviolet radiation, air oxidation, cyclic temperature and humidity, and repeated vehicle loading. Although asphalt aging does not directly induce visible pavement distress, it significantly degrades the adhesion, water stability, and crack resistance of the mixtures, which severely reduces the service life of the pavement. Accordingly, accelerated aging tests were carried out on the five asphalt mixtures in accordance with procedure T0734 in JTG 3410-2025. After aging, the high-temperature stability and moisture damage resistance were evaluated using the wheel tracking and freeze–thaw splitting tests, respectively. Three parallel specimens were tested for the wheel tracking test, while eight specimens were used for the freeze–thaw splitting test and divided equally into unconditioned and freeze–thaw-conditioned groups. The reported results represent the mean values of the corresponding replicate specimens.

## 3. Results and Discussions

### 3.1. Volumetric Properties of Asphalt Mixtures

The physical indices of the asphalt mixtures at OAC condition are presented in Table 6. Compared with AC-BA, all polymer-modified asphalt mixtures with the same AC-16 gradation exhibited improved Marshall stability, indicating that polymer modification effectively enhanced the load-bearing capacity of asphalt mixtures. Since these mixtures shared an identical aggregate gradation, the performance differences mainly originated from the modification effect of asphalt binders. Among them, AC-SMA showed the highest Marshall stability, demonstrating the effectiveness of SBS modification in improving mixture strength and deformation resistance. AC-TMA also exhibited enhanced mechanical performance compared with AC-BA, while its lower flow value suggested a stronger resistance to deformation. After introducing SMC into the TPU-modified system, AC-TSMA achieved a better balance between strength and deformation capacity, with an increased Marshall stability of 13.48 kN and a moderate flow value of 3.34 mm, indicating that the composite modification improved the adaptability of the polymer-modified binder system.

Compared with AC-TSMA, CAVF-TSMA exhibited different volumetric characteristics, with increased VMA and VFA, resulting from the skeleton-dense aggregate structure designed by the CAVF method. The enhanced void structure provides more space for asphalt mastic distribution and promotes the formation of a stable coarse aggregate skeleton. Although its Marshall stability was lower than that of AC-TSMA under short-term loading conditions, the CAVF structure may contribute to improved structural stability under long-term loading conditions. These results indicated that polymer modification primarily improves binder-related mechanical properties, while CAVF gradation optimization regulates the internal skeleton structure, and their combination provides a potential strategy for developing durable asphalt mixtures.

### 3.2. Pavement Performance of Asphalt Mixtures

#### 3.2.1. High-Temperature Stability

**Figure 12:**
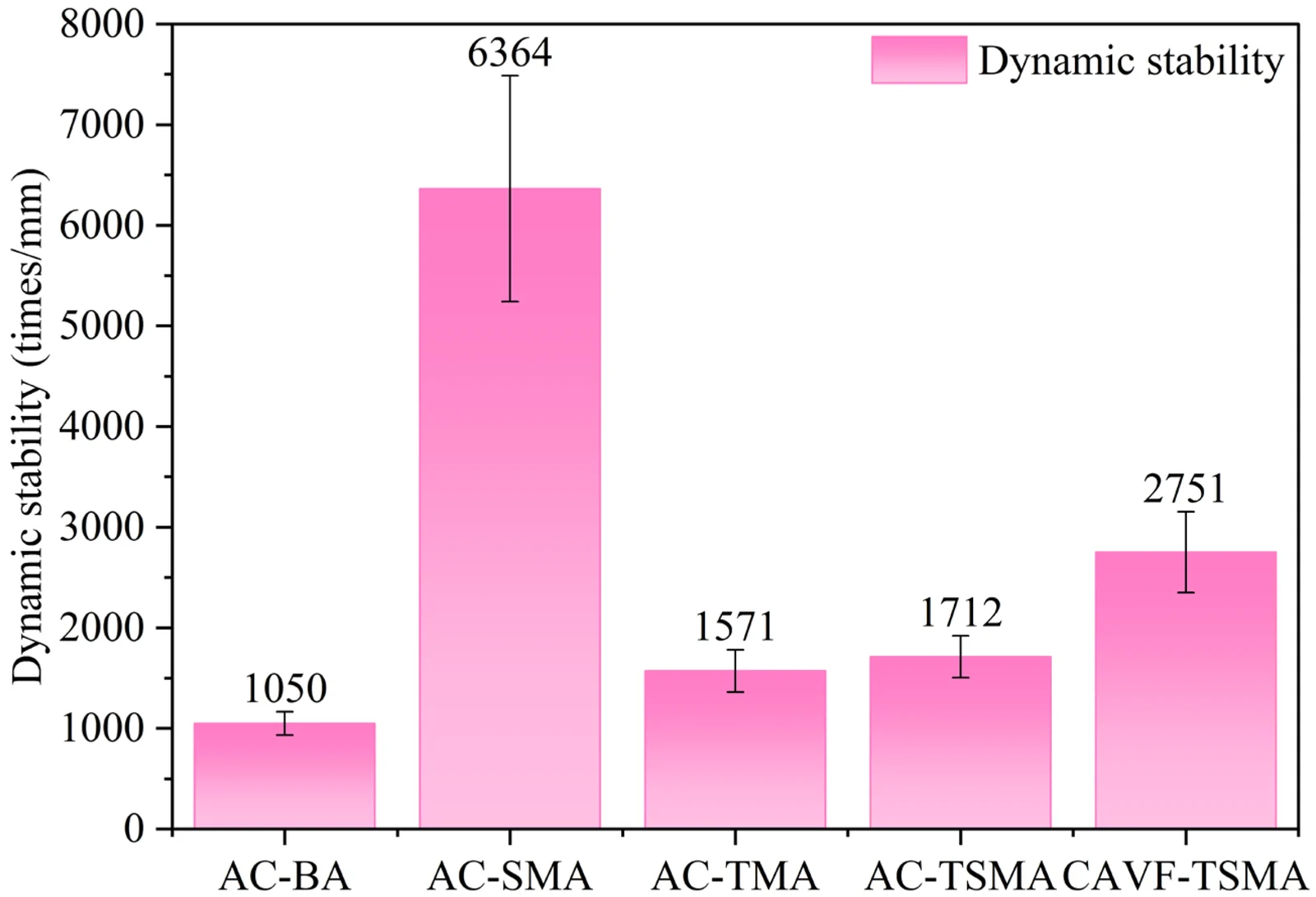
Dynamic stability of five types of asphalt mixtures.

The dynamic stability results for the various asphalt mixtures are presented in Figure 12. The AC-BA mixture exhibited a dynamic stability of 1050 times/mm, indicating limited resistance to permanent deformation under high-temperature loading. After polymer modification, all modified asphalt mixtures showed improved rutting resistance, demonstrating the effectiveness of polymer modification in enhancing the high-temperature stability of asphalt mixtures.

Among the AC-graded mixtures, AC-SMA exhibited the highest dynamic stability of 6364 times/mm, which was significantly higher than that of AC-BA. This improvement was attributed to the excellent high-temperature stability of SBS-modified asphalt, where the elastic polymer network enhanced binder cohesion and restricted the viscous flow of asphalt under elevated temperatures. Compared with AC-BA, TPU modification increased the dynamic stability of AC-TMA to 1571 times/mm, indicating improved deformation resistance. After incorporating SMC into the TPU-modified system, the dynamic stability of AC-TSMA further increased to 1712 times/mm, suggesting that SMC modification improved the high-temperature stability of the TPU-based binder system by enhancing its resistance to deformation.

Compared with AC-TSMA, the CAVF-TSMA mixture exhibited a further increase in dynamic stability to 2751 times/mm, despite using the same modified binder. This improvement indicates that the optimized coarse aggregate skeleton contributed significantly to rutting resistance. The CAVF gradation promotes stronger coarse aggregate interlocking and improves stress transfer within the mixture, thereby reducing permanent deformation during high-temperature loading. Although its dynamic stability remained lower than that of AC-SMA, the combination of CAVF skeleton design and TSMA modification provided a more balanced approach by enhancing structural stability while maintaining the advantages of polymer modification. Overall, polymer modification primarily improves binder-related deformation resistance, whereas CAVF gradation optimization further enhances the mechanical stability of the TSMA-modified mixture through aggregate skeleton improvement.

#### 3.2.2. Low-Temperature Crack Resistance

**Figure 13:**
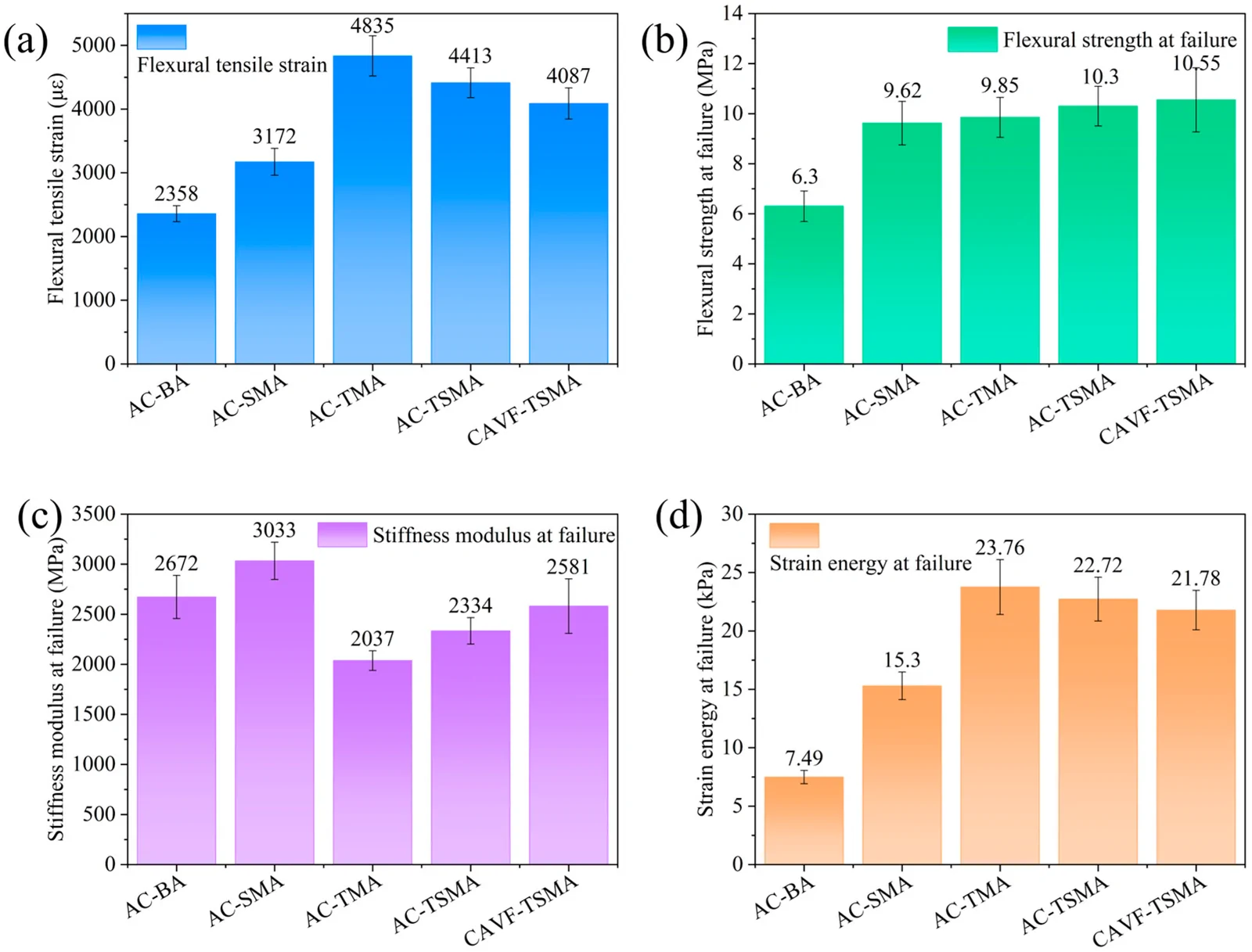
Results of low-temperature flexural tests for five types of asphalt mixtures ((**a**), flexural strain; (**b**), failure strength; (**c**), stiffness failure modulus; (**d**), failure strain energy).

Figure 13 presents the low-temperature bending test results of the five asphalt mixtures. The results indicate that AC-BA exhibited the weakest low-temperature performance, with a failure strain of 2358 με and a flexural strength at failure of 6.30 MPa, while its stiffness modulus at failure reached 2672 MPa. Compared with AC-BA, AC-SMA exhibited higher failure strain, flexural strength, and strain energy at failure, indicating improved low-temperature deformation capacity after SBS modification. Moreover, AC-SMA also showed the highest stiffness modulus among the modified mixtures, with a value of 3033 MPa.

Compared with AC-SMA, the failure strains of AC-TMA and AC-TSMA increased by 52% and 39%, respectively, while their stiffness moduli decreased by 33% and 23%, respectively. Meanwhile, both mixtures exhibited higher flexural strength and strain energy at failure than AC-SMA. These results indicate that TPU modification improved the low-temperature flexibility and deformation tolerance of asphalt mixtures while maintaining sufficient load-bearing capacity. This improvement can be attributed to the elastic characteristics of TPU. As an elastic polymer material, TPU can form a flexible polymer network within the asphalt matrix, which improves the deformation capacity of the mixture under low-temperature loading.

After SMC was incorporated into the TPU system, the grafted SMC segments improved the interaction and compatibility between the polymer phase and asphalt while maintaining the flexibility provided by TPU soft segments. Compared with AC-TMA, the failure strain and strain energy at failure of AC-TSMA decreased slightly by approximately 9% and 4%, respectively, indicating a minor reduction in low-temperature deformation capacity. However, considering its enhanced high-temperature performance, AC-TSMA achieved a better balance between high-temperature stability and low-temperature cracking resistance compared with AC-TMA.

Compared with AC-TSMA, CAVF-TSMA exhibited slightly reduced failure strain and strain energy at failure, while its flexural strength and stiffness modulus increased by approximately 2% and 11%, respectively. This indicates that the CAVF aggregate structure improved the load-bearing capacity and structural stability of the mixture while maintaining acceptable low-temperature deformation performance. This improvement may be associated with the optimized coarse aggregate skeleton formed by CAVF gradation, which enhanced aggregate contact and stress transfer within the mixture. Overall, CAVF-TSMA exhibited a favorable balance among deformation capacity, mechanical strength, and structural stability.

#### 3.2.3. Moisture Damage Resistance

**Figure 14:**
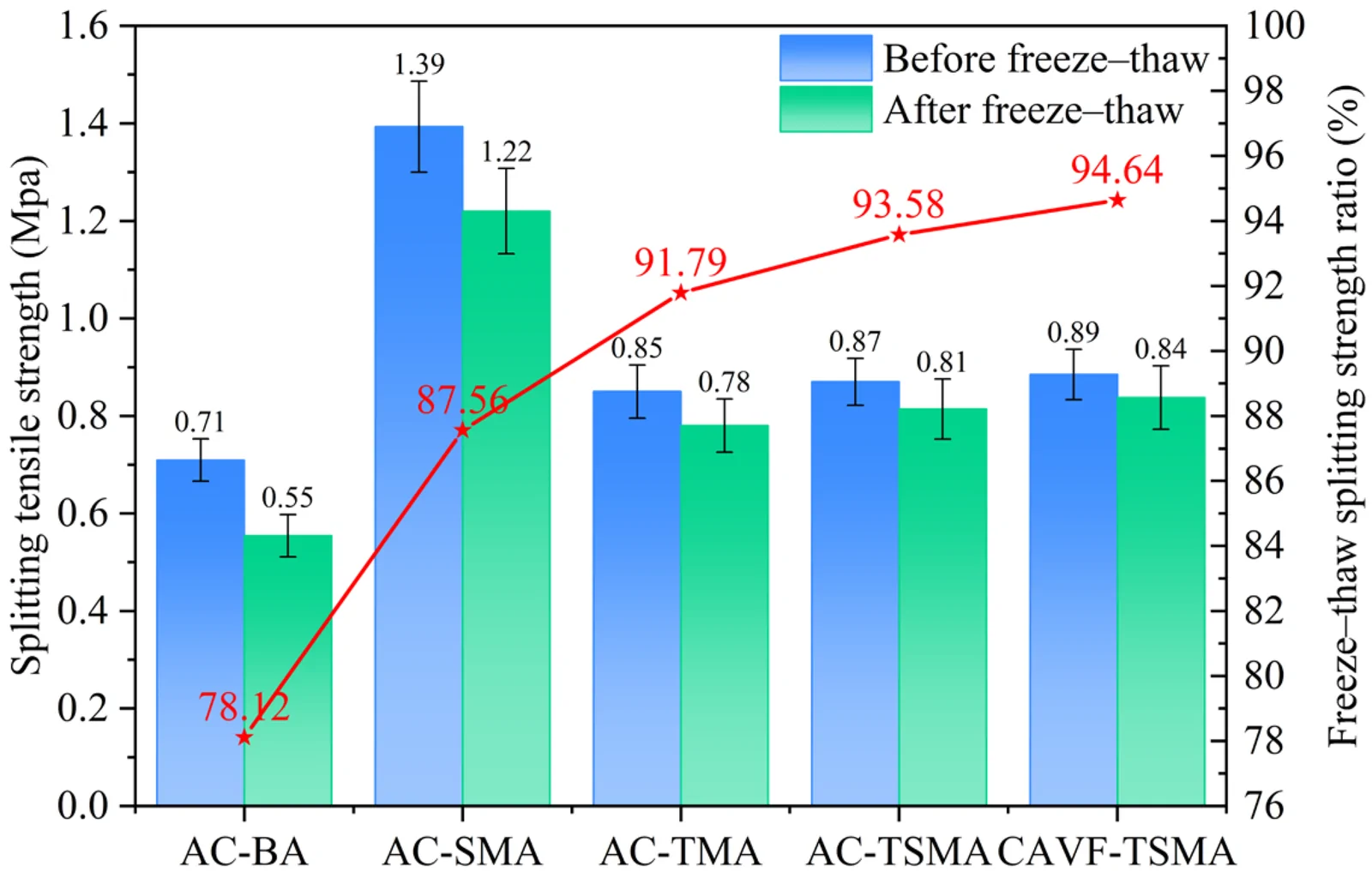
Freeze–thaw splitting test results of five asphalt mixtures.

Figure 14 presents the freeze–thaw splitting test results of the five asphalt mixtures. Differences in moisture damage resistance were observed among mixtures with different binders and gradation designs. A tensile strength ratio (TSR) of only 78.12% was recorded for AC-BA, indicating the poorest moisture damage resistance. Interfacial stripping between asphalt and aggregates was prone to occur under freeze–thaw cycling. Although AC-SMA featured relatively high splitting tensile strength and prominent mechanical performance, its TSR was 87.56%. Its moisture damage resistance was weaker than that of AC-TMA, AC-TSMA, and CAVF-TSMA mixtures. Compared with that of AC-TMA, the TSR of AC-TSMA was further increased to 93.58%, with better moisture damage resistance. This improvement was mainly attributed to the maleic anhydride-grafted structure of SMC, which enhanced the interfacial bonding strength between asphalt and aggregates and effectively inhibited adhesion failure caused by moisture intrusion. In contrast, the highest TSR of 94.64% was achieved by CAVF-TSMA with the CAVF skeleton-dense gradation design, which exhibited optimal moisture damage resistance. It inherited the excellent interfacial bonding properties of the SMC-modified system on the one hand. On the other hand, the skeleton-dense structure formed by the CAVF method reduced freeze–thaw damage paths. Overall, the most outstanding performance in moisture damage resistance was presented by the CAVF-TSMA mixture. Obvious advantages were observed for AC-TSMA over AC-TMA. Although AC-SMA had relatively high tensile strength, its moisture damage resistance was still inferior to those of the TPU-modified asphalt mixtures.

#### 3.2.4. Fatigue Resistance

**Figure 15:**
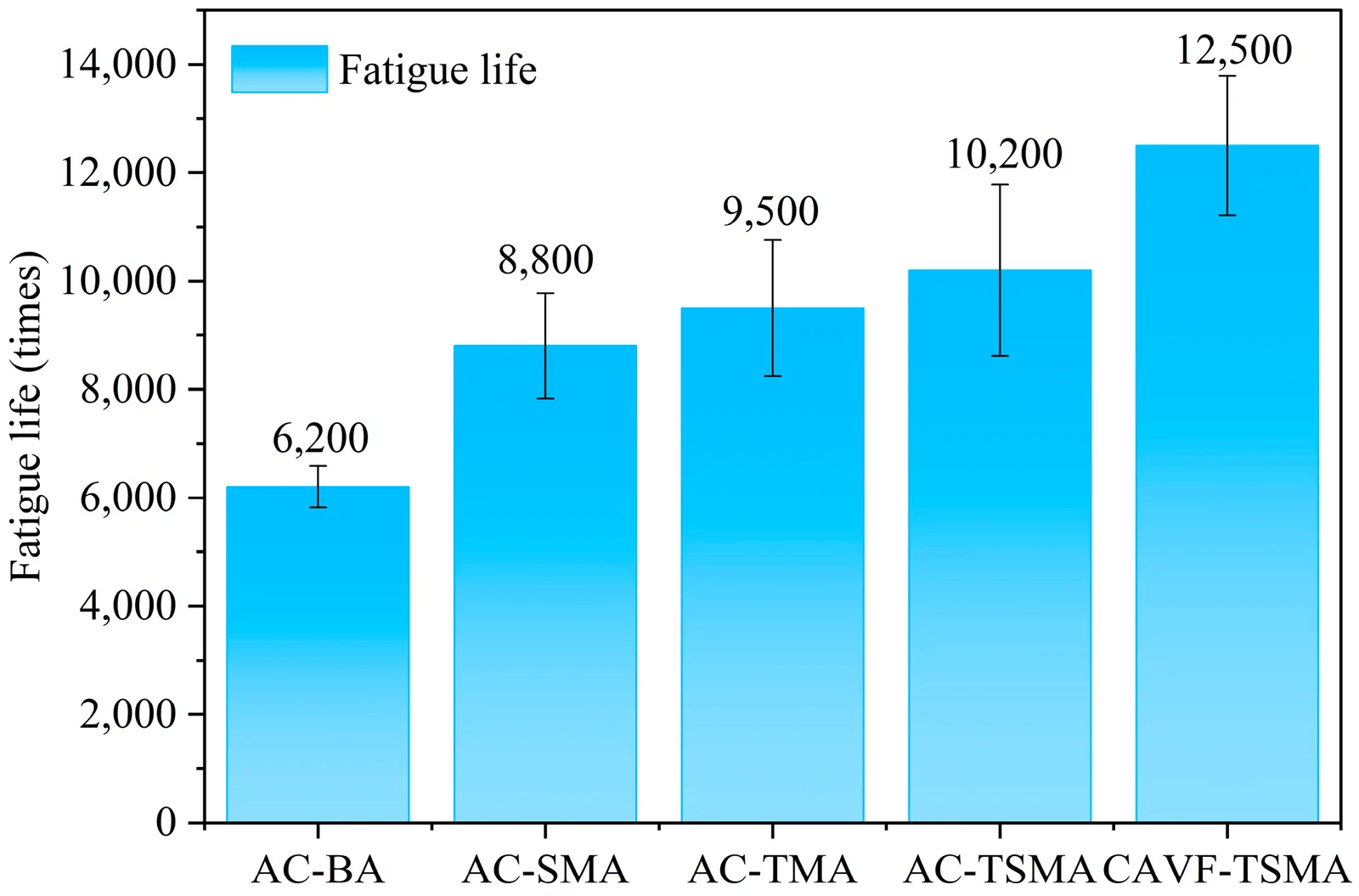
Test results of fatigue life of five types of asphalt mixtures.

Figure 15 presents the fatigue life test results of the five asphalt mixtures. A fatigue life of only 6200 cycles was recorded for AC-BA, which was most prone to fatigue crack initiation and failure under cyclic loading. The fatigue life of AC-SMA was increased to 8800 cycles, which reflected that SBS modification could improve the fatigue resistance of the mixture to a certain extent by enhancing the toughness of the binder. The mean fatigue lives of AC-TMA and AC-TSMA reached 9500 and 10,200 cycles, respectively, both higher than the mean value of the SBS-modified mixture. These improvements were mainly attributed to the TPU elastic phase, which could construct a continuous elastic network in the asphalt matrix, effectively dissipate fatigue stress under cyclic loading, and inhibit crack initiation and propagation. After SMC graft modification, the interfacial bonding strength between the binder and aggregates was strengthened, and the self-healing ability of the system was improved to some extent, which slowed down the accumulation rate of fatigue damage. Accordingly, the fatigue performance of AC-TSMA was further optimized compared with that of AC-TMA.

When AC-TSMA and CAVF-TSMA were compared under the same modified binder condition, the mean fatigue life increased from 10,200 to 12,500 cycles, corresponding to an increase of approximately 22.5%. This was because coarse aggregates in the CAVF gradation formed a stable interlocking skeleton structure, which led to more uniform internal stress distribution in the mixture and effectively reduced the degree of local stress concentration. Meanwhile, the dense gradation feature reduced internal void defects and extended the propagation path of fatigue cracks, thereby improving the long-term fatigue service performance of the mixture. Overall, the CAVF-TSMA mixture combined the high toughness of the modified binder and the structural advantages of the skeleton gradation, and exhibited optimal fatigue performance, which could effectively extend the service life of asphalt pavements.

### 3.3. Aging Resistance of Asphalt Mixtures

**Figure 16:**
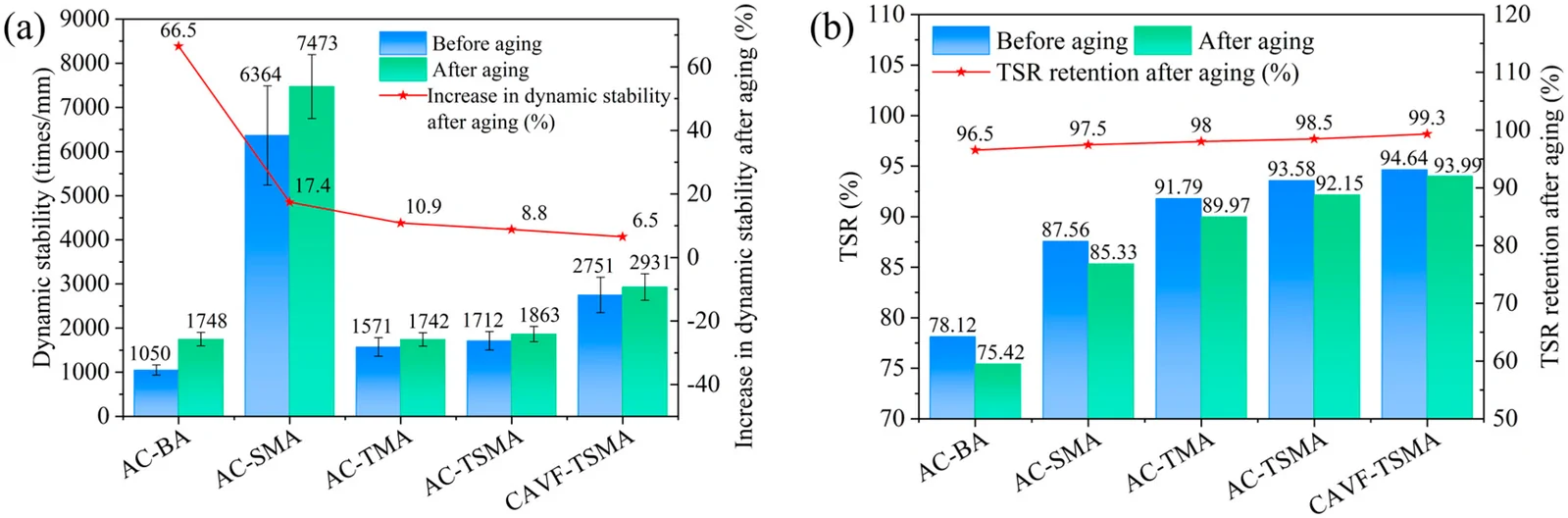
Results of aging resistance tests for five types of asphalt mixtures ((**a**), dynamic stability; (**b**), TSR).

Figure 16 presents the test results of performance changes before and after aging for the five asphalt mixtures. It was derived from the results that dynamic stability increased to varying degrees and TSR decreased to different extents for all five mixtures after aging. The increase rates of dynamic stability ranged from 6.5% to 66.5%. The reduction rates of TSR ranged from 0.7% to 3.5%. The core reason for the universal increase in dynamic stability was that thermo-oxidative aging induced oxidative polycondensation of the asphalt binder, which increased hardness and viscosity. The overall stiffness of the mixture was elevated, followed by enhanced high-temperature deformation resistance, reduced flexibility, and weakened moisture damage resistance. Among the five mixtures, both the increase in dynamic stability and the decrease in TSR were the largest for AC-BA. Its dynamic stability rose from 1050 times/mm to 1748 times/mm, with an increase rate of 66.5%, and its TSR declined from 78.12% to 75.42%, with a reduction rate of 3.5%. This indicated the poorest aging resistance. The reason was that the initial viscosity of its binder was low and the hardening effect was most prominent, which resulted in the largest increase in dynamic stability and the largest decrease in TSR. The overall aging resistance of AC-SMA was better than that of AC-BA, with a dynamic stability increase of approximately 17.4% and a TSR decrease of about 2.5%. However, AC-SMA exhibited greater aging-induced changes than AC-TMA and AC-TSMA. This was because SMA exhibited high sensitivity to thermo-oxidative, ultraviolet, and hygrothermal coupling environments, and was prone to deterioration characteristics such as hardening and elasticity attenuation after aging. Nevertheless, by constructing an elastic network structure, it could delay asphalt oxidation and component degradation to a certain extent, which made indices such as TSR and dynamic stability retention rate after long-term aging higher than those of BA.

Under the same aging condition, TPU in AC-TMA and AC-TSMA can interact with polar components in asphalt through urethane-related chemical bonding and network formation, which helps retard oxidative hardening and maintain the structural integrity of the modified binder during aging [34,35]. Previous FTIR and microscopic studies have shown that TPU incorporation can suppress the formation of aging-related oxygen-containing groups and improve the microstructural stability of modified asphalt, thereby contributing to improved aging resistance [15]. Corresponding test results showed that AC-TMA had a dynamic stability increase of approximately 10.9% and a TSR decrease of about 2.0%, while AC-TSMA had a dynamic stability increase of about 8.8% and a TSR decrease of approximately 1.5%. The performance attenuation degree of the composite modified system was lower than those of the single modified system and the base asphalt system. On this basis, SMC further formed a synergistic stabilization system with TPU, which enhanced the overall stiffness and interfacial bonding strength of the asphalt binder and effectively inhibited performance deterioration caused by aging.

Obvious differences in aging resistance were observed for TPU/SMC-modified asphalt mixtures with CAVF and AC gradations. The CAVF gradation could form a more stable aggregate interlocking structure with uniform mineral void distribution. Its high-temperature performance was mainly borne by the aggregate skeleton and was least affected by asphalt binder aging, which enabled the mixture to maintain good deformation capacity and structural stability after long-term aging. In comparison, the binder distribution within the AC gradation was relatively concentrated, which was more prone to stress concentration under the effect of aging and resulted in obvious attenuation of pavement performance. Under the same aging condition, the dynamic stability of CAVF-TSMA increased by only 6.5%, and its TSR decreased by only 0.7%. The aging variation amplitudes of both indices were smaller than those of AC-TSMA, which had an 8.8% increase in dynamic stability and a 1.5% decrease in TSR. This indicated that compared with the AC gradation, the CAVF gradation had obvious advantages in improving the aging resistance of TPU/SMC-modified asphalt mixtures.

## 4. Conclusions

Based on the performance of TPU-modified and TPU/SMC composite-modified asphalt mixtures, the regulatory effects of in situ prepolymerization modification and graft modification on the mechanical properties, pavement performance, and aging resistance of the mixtures were clarified. By comparing AC-TSMA and CAVF-TSMA mixtures, the effect of CAVF-based aggregate skeleton optimization on the pavement performance of TSMA mixtures was evaluated. The main research conclusions were drawn as follows.

(1) AC-TSMA exhibited the lowest VMA among the AC-graded polymer-modified mixtures while maintaining relatively high VFA and Marshall stability and a moderate flow value, indicating favorable volumetric and mechanical characteristics. In contrast, CAVF-TSMA exhibited a coarse aggregate skeleton packing structure, accompanied by higher VMA and VFA.
(2) TPU/SMC composite modification achieved synergistic optimization of both high-temperature and low-temperature properties of the mixtures. Its high-temperature dynamic stability was increased by over 60% compared with that of the base asphalt mixture, and its low-temperature crack resistance was also superior to that of the traditional SBS-modified system. CAVF aggregate skeleton optimization further improved the performance of the TSMA-modified mixture. The fatigue life of CAVF-TSMA increased by 22.5% compared with that of AC-TSMA, accompanied by improvements in high-temperature stability and moisture damage resistance.
(3) Under the applied laboratory aging condition, TPU-modified asphalt mixtures exhibited lower aging sensitivity than conventional AC-SMA, benefiting from the relatively stable molecular structure of TPU. The dynamic stability of AC-TSMA increased by only 8.8%, while its TSR retention reached 98.5%. CAVF gradation further reduced the variation in dynamic stability to 6.5% and increased the TSR retention to 99.3%.

## Figures and Tables

**Table 1:** Technical properties of asphalt binders.

Testing Parameters	Unit	BA	SMA	ITMA	TSMA	Test Methods
25 °C penetration	dmm	88.2	74.7	75.4	75.2	JTG 3410-T 0604
Ductility	cm	80 (10 °C)	37.4 (5 °C)	6.88 (5 °C)	7.82 (5 °C)	JTG 3410-T 0605
Softening point (R&B)	°C	45.4	78.3	51.3	54.1	JTG 3410-T 0606

**Table 2:** Technical properties of coarse aggregates.

Items	Unit	Particle Size Range (mm)				Specification Requirement
		3~6	4.75~9.5	9.5~13.2	13.2~19	
Bulk specific gravity (BSG)	-	2.813	2.696	2.692	2.696	-
Apparent specific gravity	-	2.927	2.757	2.746	2.739	-
Water absorption	%	1.39	0.3	0.1	0.1	≤2
<0.075 mm	%	1.23	0.3	0.2	0.2	≤2
Flat and elongated particle content	%	5	6	7.8	8.2	-
Aggregate crushing value	%	-	-	11.4	16.4	≤22
Los Angeles abrasion loss	%	-	-	22.7	20.3	≤30

**Table 3:** Technical properties of fine aggregates.

Items	Unit	Test Value	Design Requirement
Apparent specific gravity	-	2.586	≥2.5
Sand equivalent	%	65	≥60
<0.075 mm	%	1.23	≤10

**Table 4:** Technical properties of mineral filler.

Items	Unit	Test Value	Design Requirement
Apparent specific gravity	-	2.709	≥2.5
Moisture content	%	0.2	<1
<0.6 mm	%	100	100
<0.15 mm	%	100	90~100
<0.075 mm	%	95.1	75~100
Heating stability	-	No obvious color change	-
Appearance	-	Dry and free from agglomeration	Free from agglomeration

**Table 5:** The passing percentages of each aggregate fraction across all sieve sizes and the corresponding mixing proportions of each fraction.

Sieve Size (mm)	Passing Percentage (%)					
	13.2~19	9.5~13.2	4.75~9.5	3~6	Manufactured Sand	Mineral Filler
19	100	100	100	100	100	100
16	56.4	100	100	100	100	100
13.2	5.6	100	100	100	100	100
9.5	0.5	15	98.8	100	100	100
4.75	0.2	0.2	6.2	90.0	99.7	100
2.36	0.2	0.2	0.3	17.7	89.9	100
1.18	0.2	0.2	0.3	5.8	65.3	100
0.6	0.2	0.2	0.3	3.5	46.4	100
0.3	0.2	0.2	0.3	2.9	28	100
0.15	0.2	0.2	0.3	1.7	19.4	100
0.075	0.2	0.2	0.3	1.2	13.9	95.1
0	0	0	0	0	0	0
Proportion	20%	15%	22%	10%	29%	4%

**Table 6:** Physical indices of five asphalt mixtures at OAC condition.

Types	BSG	VV	VMA	VFA	MS	FL
		(%)	(%)	(%)	(KN)	(mm)
AC-BA	2.395	3.82	15.41	75.20	12.78	3.86
AC-SMA	2.409	4.15	14.06	70.45	14.42	3.95
AC-TMA	2.410	4.30	14.00	69.33	13.32	2.14
AC-TSMA	2.416	4.04	13.78	70.66	13.48	3.34
CAVF-TSMA	2.394	4.26	15.75	73.00	10.56	2.84

## Data Availability

The original contributions presented in this study are included in the article. Further inquiries can be directed to the corresponding authors.
